# Leber's hereditary optic neuropathy–associated ND1 3733G>C mutation ameliorates the mitochondrial quality control and cellular homeostasis

**DOI:** 10.1016/j.jbc.2025.110464

**Published:** 2025-07-08

**Authors:** Meiheriayi Yasheng, Yanchun Ji, Yunfan He, Qiuzi Yi, Huanhuan Zhang, Wenqi Shan, Kai Wang, Juanjuan Zhang, Ya Li, Feilong Meng, Minglian Zhang, Jun Qin Mo, Shihui Wei, Min-Xin Guan

**Affiliations:** 1 Center for Mitochondrial Biomedicine and Department of Ophthalmology, the Fourth Affiliated Hospital, Zhejiang University School of Medicine, Yiwu, Zhejiang, China; 2 Center for Genetic Medicine, Zhejiang University International Institute of Medicine and School of Medicine, Yiwu, Zhejiang, China; 3 Institute of Genetics, Zhejiang University, Hangzhou, Zhejiang, China; 4 Department of Genetics and Metabolic Diseases and Division of Medical Genetics and Genomics, the Children's Hospital, Zhejiang University School of Medicine, National Clinical Research Center for Child Health, Hangzhou, Zhejiang, China; 5 School of Ophthalmology and Optometry, Wenzhou Medical University, Wenzhou, Zhejiang, China; 6 Department of Ophthalmology, Hebei Provincial Eye Hospital, Xingtai, Hebei, China; 7 Department of Pathology, Rady Children’s Hospital, University of California at San Diego, San Diego, California, USA; 8 Department of Ophthalmology, Chinese PLA General Hospital, Beijing, China

**Keywords:** Leber's hereditary optic neuropathy, mitochondria, ND1 mutation, oxidative phosphorylation, vision loss, NADH:ubiquinone oxidoreductase, ATP, reactive oxygen species, mitophagy, apoptosis, autophagy, mitochondrial fusion and fission

## Abstract

Leber’s hereditary optic neuropathy (LHON) is a paradigm for mitochondrial retinopathy because of mitochondrial DNA (mtDNA) mutations. However, the mechanism underlying LHON-linked mtDNA mutations, especially their impact on mitochondrial and cellular integrity, is not well understood. Recently, the ND1 3733G>C (p.E143Q) mutation was identified in three Chinese pedigrees with LHON. In this study, we investigated the pathogenic mechanism of m.3733G>C mutation using cybrids generated by fusing mtDNA-less *ρ*^0^ cells with enucleated cells from a Chinese patient carrying the m.3733G>C mutation and control subject. Molecular dynamics simulations showed that p.E143Q mutation destabilized these interactions between residues E143 and S110/Y114 or between S141 and W290 in the ND1. Its impact of ND1 structure and function was further evidenced by reduced levels of ND1 in mutant cells. The m.3733G>C mutation caused defective assembly and activity of complex I, respiratory deficiency, diminished mitochondrial ATP production, and increased production of reactive oxygen species in the mutant cybrids carrying the m.3733G>C mutation. These mitochondrial dysfunctions regulated mitochondrial quality control *via* mitochondrial dynamics and mitophagy. The m.3733G>C mutation–induced dysfunction yielded elevating mitochondrial localization of DRP1, decreasing network connectivity, and increasing fission with abnormal morphologies. Furthermore, the m.3733G>C mutation downregulated ubiquitin-dependent mitophagy pathway, evidenced by decreasing the levels of Parkin and PINK1, but not ubiquitin-independent mitophagy pathway. The m.3733G>C mutation–induced deficiencies reshaped the cellular homeostasis *via* impairing autophagy process and promoting intrinsic apoptosis. Our findings provide new insights into pathophysiology of LHON arising from the m.3733G>C mutation–induced mitochondrial dysfunctions and reprograming organocellular and cellular homeostasis.

Retinal ganglion cells (RGCs) are the high energy-demanding neurons, transmitting visual information from the retina to the brain. These RGCs are susceptible to degeneration defects in oxidative phosphorylation (OXPHOS) (1, 2). In particular, the degeneration of RGC because of mitochondrial dysfunctions manifests optic neuropathy, including autosomal dominant optic atrophy and Leber’s hereditary optic neuropathy (LHON) (1, 2, 3, 4, 5). LHON is the most common example of maternally inherited eye disorder due to mitochondrial DNA (mtDNA) mutations (4, 5, 6). This disorder is characterized by bilateral, painless, subacute, and central visual loss in young adults (7, 8). Numerous mtDNA mutations have been identified as contributing to the pathogenesis of LHON to various extents (6, 9, 10, 11, 12). In the majority of cases worldwide, LHON was caused by three primary mtDNA point mutations: ND1 3460G>A, ND4 11778G>A, and ND6 14484T>C, affecting the core subunits of NADH:ubiquinone oxidoreductase (complex I) (9, 10, 11, 12, 13, 14, 15). Other LHON-associated LHON mutations included the ND1 3866T>C, 3635G>A, 3394T>C, ND5 12238T>C, and tRNA^Ala^ 5587T>C mutations (16, 17, 18, 19, 20, 21, 22, 23). These LHON-associated mtDNA mutations occurring nearly homoplasmy or homoplasmy conferred reduced activity of complex I, thereby leading to decreases in ATP synthesis and increasing generation of reactive oxygen species (ROS) (21, 22, 23, 24, 25). Subsequently, the energy failure and increasing oxidative stress may be responsible for the degeneration of RGCs (1, 3, 4, 26). However, the mechanism underlying these LHON-associated mtDNA mutations, especially impact on mitochondrial function and maintain cellular integrity, is not well understood.

Most recently, we identified the ND1 3733G>C (p.E143Q) mutation in three genetically unrelated Chinese pedigrees with suggestively maternal inheritance of LHON from a large cohort of Chinese patients (15, 22, 23). The m.3733G>C mutation resulted in the substitutions of a highly conserved residue E143 to Q in the fourth transmembrane domain of ND1. Thus, we hypothesize that the m.3733G>C mutation perturbs both the structure and function of complex I. The impact of E143Q on the structure and function of ND1 was evaluated by molecular dynamics (MD) simulation, based on the crystal structure of mammalian complex I (Protein Data Bank [PDB] entry: 5XTD) (27). To further investigate the pathogenic mechanism of the m.3733G>C mutation, cybrid cell lines were constructed by transferring mitochondria from lymphoblastoid cell lines derived from an affected matrilineal relative carrying the mtDNA mutation and from a control individual belonging to the same mtDNA haplogroup but lacking the mtDNA mutation, into human mtDNA-less (*ρ*^0^) cells (28, 29). Despite irrelevant to affected tissues, these cybrid lines, which contain the constant nuclear and mtDNA genetic backgrounds, allow us to analyze directly biochemical phenotypes because of the ND1 3733G>C mutation. These cell lines were further assessed for the effects of the m.3733G>C mutation on the assembly, enzymatic activities of electron transport chain complexes, the rate of oxygen consumption rate (OCR), mitochondrial ATP production, and ROS production. Furthermore, we assessed whether the mitochondrial dysfunctions resulting from the m.3733G>C mutation regulated mitochondrial quality control processes involving fission and fusion and mitophagy. We then examined if the m.3733G>C mutation–induced deficiencies reshaped cellular homeostasis through autophagy and intrinsic apoptosis.

## Results

### Evaluation of three Han Chinese families with suggestive maternal inheritance of LHON

**Figure 1:**
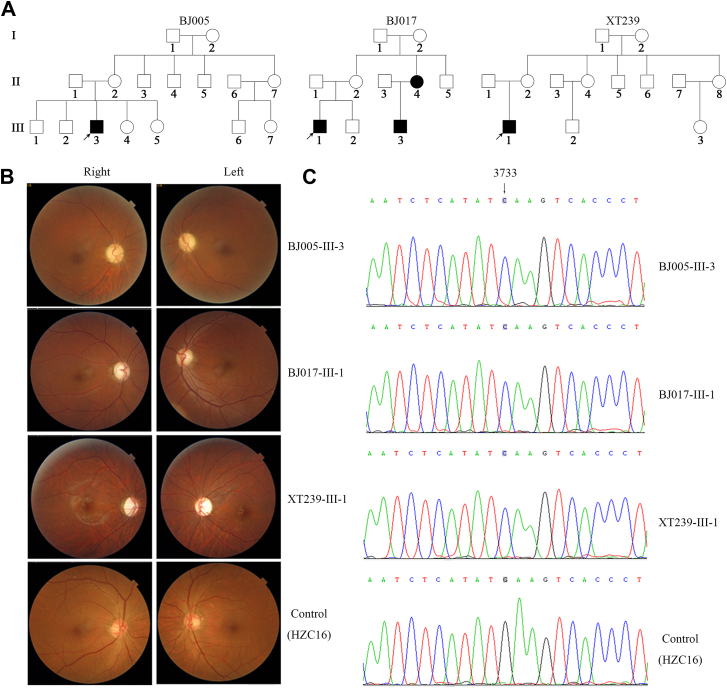
**Identification of LHON-linked ND1 3733G>C (p.E143Q) mutation.***A*, three Han Chinese pedigrees with Leber’s hereditary optic neuropathy. Vision-impaired individuals are indicated by *filled symbols*, and probands are indicated with *arrows*. *B*, fundus photographs from three probands and one control subject of three Chinese families. *C*, partial Sanger sequence chromatograms of ND1 gene from three probands and one control subject. LHON, Leber’s hereditary optic neuropathy.

The novel ND1 3733G>C mutation was identified in three genetically unrelated Chinese pedigrees among a large cohort of 1793 Chinese probands with LHON (15, 22). All available members of these pedigrees underwent comprehensive physical and ophthalmologic examinations to identify personal or family medical histories of visual impairments and other clinical abnormalities. As shown in Figure 1, *A* and *B* and Table S1, only five (four males/one female) of 39 matrilineal relatives in these families exhibited the variable severity and age at onset in optic neuropathy, whereas other members of these families had normal vision. The average age at onset of optic neuropathy in these affected matrilineal relatives ranged from 11 to 57 years, with an average of 26.6 years. The penetrances of optic neuropathy in these families varied from 7.7% to 42.9%, with an average of 20.6%. The ratios between affected male and female matrilineal relatives were 1:0, 2:1, and 1:0, respectively. Furthermore, all affected matrilineal relatives of these families revealed no other clinical abnormalities, including hearing loss, diabetes, and neurological diseases.

As shown in Figure 1*C* and Table S2, these subjects harbored the identical m.3733G>C mutation and distinct sets of mtDNA polymorphisms belonging to the Eastern Asian haplogroups G2a1, R11a, D4, respectively (30). The m.3733G>C mutation resulted in the replacement of a highly conserved glutamic acid at position 143 with glutamine (E143Q) in the ND1. Further analysis confirmed the presence of homoplasmic m.3733G>C mutation in all matrilineal relatives but not in other members of these families and 485 vision normal Chinese control subjects (data not shown). Other variants in RNAs and polypeptides were further evaluated by phylogenetic analysis of these variants and sequences from the other 16 different species and the presence of 485 Chinese controls (31). None of these variants revealed the functional significance (Table S2).

### MD simulation analysis

**Figure 2:**
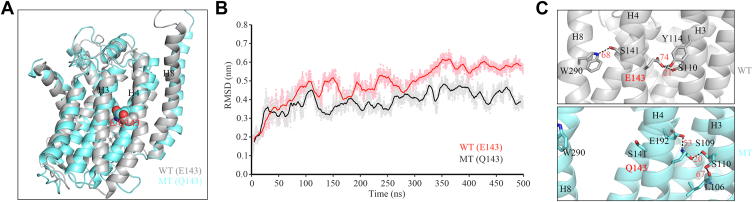
**Analysis for molecular dynamics simulations.***A*, molecular dynamics simulations on the WT and mutated (MT) ND1. Superimposition of the structures of WT (E143) (*gray*) and mutant (Q143) *(cyan*) proteins at the end of the simulations, based on the crystal structure of mammalian complex I (Protein Data Bank entry: 5XTD) (27). *B*, time evolution of the RMSD values of all backbone atoms for the WT (*black lines*) and E143Q mutant (*red lines*) proteins. *C*, the electrostatic interactions formed between E143 and neighboring residues in the WT protein (*gray*). The electrostatic interactions formed between Q143 and neighboring residues in the mutant protein (*cyan*).

The m.3733G>C (E143Q) mutation resulted in the substitutions of highly conserved residue E143 by Q in the fourth transmembrane domain of ND1. To evaluate the impact of E143Q on the structure and function of ND1, we carried out an MD simulation analysis for ND1. Based on the rational initial structure (PDB entry: 5XTD) (27), WT and mutated ND1 were evaluated by 500 ns all-atom MD simulation. As shown in Figure 2*A*, the superimposed structure of WT (E143) (*gray*) and mutant (Q143) (*cyan*) ND1 revealed that the H4 and surrounding helices H3 and H8 have undergone significant structural changes. The deviation of each structure from the initial structure during simulations was quantified by calculating the RMSD values of the two systems. As shown in Figure 2*B*, the E143Q mutant molecule exhibited a higher RMSD value from the starting conformation, as compared with the WT counterpart after 80 ns, suggesting that the Q143 substitution induced greater structural deviation in ND1 relative to the initial structure. We further analyzed the consequence of E143Q mutation on residue interactions within the ND1 structure. As shown in Figure 2*C*, there are the interactions between residues E143 and S110 and Y114 or between S141 and W290 in WT ND1 structure. The substitution of glutamic acid (E143) with glutamine (Q143) resulted in the replacement of a carboxyl group with an amide group and destabilized these interactions between residues E143 and S110/Y114 or between S141 and W290. Strikingly, the alterations introduced the new interactions between Q143 and E192/S109 in the ND1. These suggest that the E143Q mutation impacted the structure and function of ND1.

### The m.3733G>C mutation caused the decrease of ND1 level

**Figure 3:**
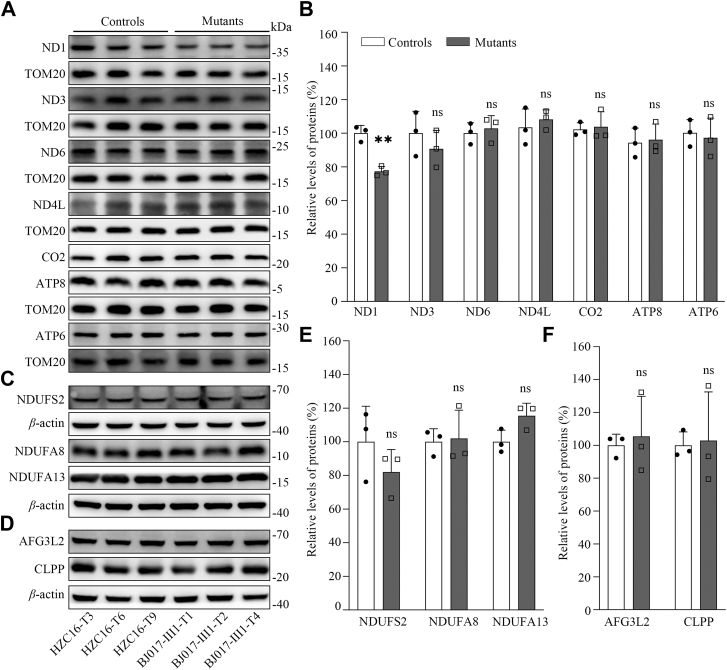
**Western blot analysis of mitochondrial proteins.***A*, Western blot analysis of mtDNA-encoding ND1, ND3, ND6, ND4L, CO2, ATP8, and ATP6 in the cybrids with TOM20 as a loading control. *B*, quantification of ND1, ND3, ND6, ND4L, CO2, ATP8, and ATP6 in mutant and control cybrids. *C* and *D*, Western blot analysis of nucleus encoding complex I subunits (NDUFS2, NDUFA8, and NDUFA13) and proteins involved in proteostasis stress (CLPP and AFG3L2) in six cell lines with *β*-actin as a loading control. *E* and *F*, quantification of NDUFS2, NDUFA8, NDUFA13, CLPP, and AFG3L2 in mutant and control cybrids. The calculations were based on three independent determinations in each cell line. The error bars indicate two standard errors of the means. *P* indicates the significance, according to the Student's *t* test of the differences between mutant and control cell lines. mtDNA, mitochondrial DNA.

We measured the levels of ND1 protein by a Western blot analysis in the mutant cell lines carrying the m.3733G>C mutation and control cell lines lacking the mutation. As shown in Figure 3, *A* and *B*, the levels of ND1 in mutant cell lines ranged from 74.8% to 80.5%, with an average of 77.3%, relative to the average values of control cell lines. By contrast, the levels of other mtDNA-encoding ND3, ND6, ND4L, CO2, ATP8, and ATP6 in the mutant cell lines were comparable with those in the control cybrids.

To examine whether m.3733G>C mutation affected the expression of nucleus-encoding subunits of complex I, we measured the levels of NDUFAS2, NDUFA8, and NDUFA13 by Western blot analysis among mutant and control cell lines. As shown in Figure 3, *C* and *E*, the levels of these subunits in the mutant cybrids were comparable with those in the control cybrids.

To assess whether the m.3733G>C mutation affected the mitochondrial homeostasis, we measured the levels of caseinolytic mitochondrial matrix peptidase proteolytic subunit (CLPP) involved in mitoribosome assembly and AFG3-like matrix AAA protease stress in mutant and control cybrids (32, 33). As shown in Figure 3, *D* and *F*, there were no significant differences in the levels of AFG3L2 and CLPP between mutant and control cybrids. These data indicated that the m.3733G>C mutation may not affect the proteostasis stress.

### Altered assembly and activity of complex I

**Figure 4:**
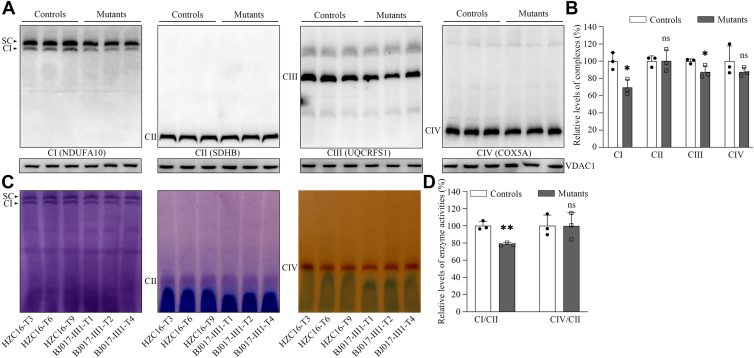
**Defective stability and activity of complex I.***A*, the levels of complexes I, II, III, and IV by BN-PAGE. About 20 μg of mitochondrial proteins from various cell lines were electrophoresed through a BN-PAGE, electroblotted, and hybridized with antibodies specific for subunits of complexes I, II, III, and IV (NDUFA10 antibody for complex I, SDHB antibody for complex II, UQCRFS1 antibody for complex III, and COX5A antibody for complex IV), and with VDAC1 as a loading control. SC, supercomplex. *B*, quantification of levels of complexes I, II, III, and IV. *C*, in-gel activity of complexes I, II, and IV. The activities of OXPHOS complexes from various cell lines after BN-PAGE were measured in the presence of specific substrates (NADH and NTB for complex I; sodium succinate, phenazine methosulfate, and NTB for complex II; DAB and cytochrome *c* for complex IV). *D*, quantification of in-gel activities of complexes I and IV. The calculations were based on three independent determinations in each cell line. The calculations were based on three independent determinations in each cell line. Graph details and symbols are explained in the legend to Figure 3. BN-PAGE, blue native-PAGE; DAB, 3,3'-diaminobenzidine; OXPHOS, oxidative phosphorylation.

We analyzed the consequence of m.3733G>C mutation on the stability and activity of complex I using the in-gel activity assay. Mitochondrial membrane proteins isolated from mutant and control cell lines were separated by blue native-PAGE (BN-PAGE), electroblotting, and hybridizing with NDUFA10 (subunit of complex I), SDHB (subunit of complex II), UQCRFS1 (subunit of complex III), COX5A (subunit of complex IV), and VDAC1 as a loading control, respectively (34, 35, 36). As illustrated in Figure 4*A*, the cybrids bearing the m.3733G>C mutation exhibited the instability of intact supercomplexes and complexes I, as compared with those in control cybrids. As shown in Figure 4*B*, the levels of complexes I, II, III, and IV in the mutant cybrids were 69.6%, 100.3%, 87.5%, and 87.6% of those average values in control cybrids, respectively. These results indicated that the m.3733G>C mutation affected the assembly of complexes I and III, respectively.

Furthermore, we assessed the consequence of m.3733G>C mutation on the activity of complexes I, II, and IV using the in-gel activity assay (36). Mitochondrial membrane proteins isolated from various cell lines were separated by BN-PAGE and stained in the presence of specific substrates of OXPHOS complexes (NADH and NTB for complex I; sodium succinate, phenazine methosulfate, and NTB for complex II; and 3,3′-diaminobenzidine and cytochrome *c* for complex IV (36)). As shown in Figure 4, *C* and *D*, defective assembly of complexes I was further confirmed in the mutant cell lines, as compared with control cell lines. In particular, the in-gel activities of complex I in mutant cell lines were 78.5%, relative to the average values of control cybrids, whereas the in-gel activities of complexes II and IV in the mutant cybrids were comparable with those of control cell lines, respectively. These data indicated the impact of m.3733G>C mutation on stability and activity of complex I.

### Deficient OXPHOS

**Figure 5:**
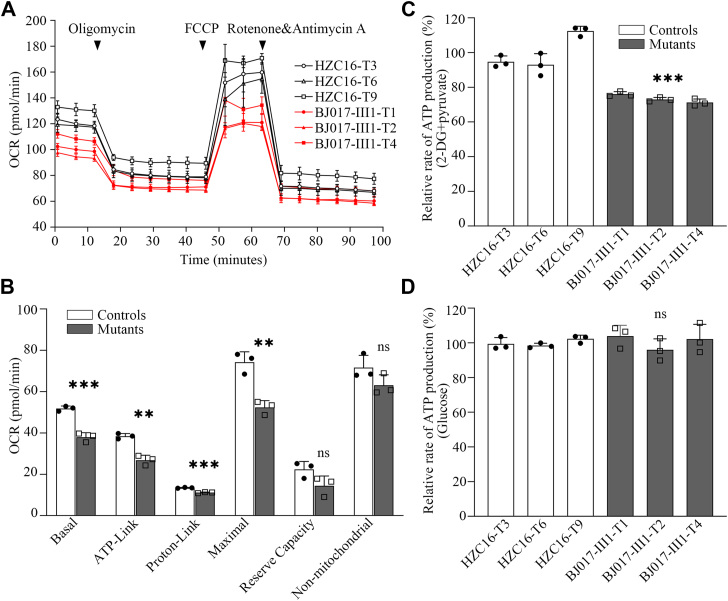
**Deficient oxidative phosphorylation.***A*, an analysis of O_2_ consumption in the various cell lines using different inhibitors. The rates of O_2_ (OCR) were first measured on 2 × 10^4^ cells of each cell line under basal condition and then sequentially added to oligomycin (1.5 mM), carbonyl cyanide p-(trifluoromethoxy) phenylhydrazone (FCCP) (0.5 mM), rotenone (1 mM), and antimycin A (1 mM) at indicated times to determine different parameters of mitochondrial functions. *B*, graphs presented the ATP-linked OCR, proton-linked OCR, maximal OCR, reserve capacity, and nonmitochondrial OCR in mutant and control cell lines. Nonmitochondrial OCR was determined as the OCR after rotenone/antimycin A treatment. Basal OCR was determined as OCR before oligomycin minus OCR after rotenone/antimycin A. ATP-lined OCR was determined as OCR before oligomycin minus OCR after oligomycin. Proton linked was determined as basal OCR minus ATP-linked OCR. Maximal was determined as the OCR after FCCP minus nonmitochondrial OCR. Reserve capacity was defined as the difference between maximal OCR after FCCP minus basal OCR. The average values of three determinations for each cell line were shown, the *horizontal dashed lines* represent the average value for each group. *C* and *D*, measurement of cellular and mitochondrial ATP levels. ATP levels from mutant and control cell lines were measured using a luciferin/luciferase assay. Mutant and control cell lines were incubated with 10 mM glucose or 5 mM 2-deoxy-d-glucose plus 5 mM pyruvate to determine ATP generation under mitochondrial ATP synthesis. Average rates of ATP level per cell line in mitochondria are shown: (*C*) ATP level in mitochondria; (*D*) ATP level in total cells. Three determinations were made for each cell line. The average values of three determinations for each cell line were shown. Graph details and symbols are explained in the legend to Figure 3. OCR, oxygen consumption rate.

OCR is an indicator of mitochondrial respiration (37). We measured the OCRs of various cell lines, including basal respiration, O_2_ consumption attributing to ATP production, proton leak, maximum respiratory rate, reserve capacity, and nonmitochondrial respiration of various mutant and control cybrid cell lines with a Seahorse Bioscience XF-96 extracellular flux analyzer (Seahorse Bioscience) (37, 38). As shown in Figure 5, *A* and *B*, the basal OCR in three mutant cybrids carrying the m.3733G>C mutation was 73.2% of the mean value measured in the control cybrids. To assess which of the enzyme complexes of the respiratory chain was perturbed in the mutant cell lines, OCRs were measured after the sequential addition of oligomycin (inhibit the ATP synthase), carbonyl cyanide p-(trifluoromethoxy) phenylhydrazone (to uncouple the mitochondrial inner membrane and allow for maximum electron flux through the electron transport chain), rotenone (to inhibit complex I), and antimycin (to inhibit complex III). The difference between the basal OCR and the drug-insensitive OCR yields the amount of ATP-linked OCR, proton-linked OCR, maximal OCR, reserve capacity, and nonmitochondrial OCR. As shown in Figure 5*B*, ATP-linked OCR, proton-linked OCR, maximal OCR, reserve capacity, and nonmitochondrial OCR in three mutant cybrids carrying m.3733G>C mutation were 69.8%, 82.8%, 70.6%, 64.3%, and 88% of the mean values measured in three control cybrids, respectively.

To assess the capacity of OXPHOS, we measured the levels of cellular and mitochondrial ATP production using a luciferin/luciferase assay (38). Populations of cells were incubated in the media in the presence of glucose and 2-deoxy-d-glucose with pyruvate to inhibit the glycolysis. As shown in Figure 5, *C* and *D*, the levels of mitochondrial ATP production in three mutant cybrids ranged from 71.2% to 75.9%, with an average of 73.3% of the average values measured in three control cybrids, whereas the levels of cellular ATP production (glucose) in mutant were comparable with those measured in the control cell lines.

### The increase of ROS production

**Figure 6:**
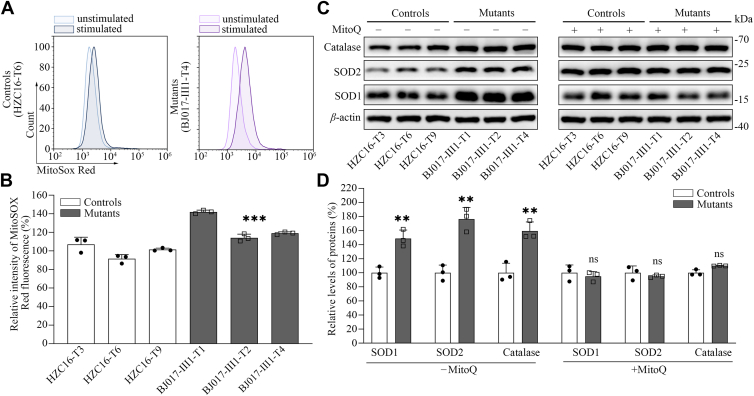
**Measurement of mitochondrial ROS**. The rates of ROS generation in the mitochondria from mutant and control cell lines were analyzed by NovoCyte flow cytometer (ACEA Biosciences) using the mitochondrial superoxide indicator MitoSOX Red (5 mM). *A*, flow cytometry histogram showing MitoSOX Red fluorescence of various cell lines. *B*, relative ratios of MitoSOX Red fluorescence intensity. The average of three determinations for each cell line is shown. *C*, Western blot analysis of three antioxidative enzymes (SOD1, SOD2, and catalase) in the presence and absence of 100 nM MitoQ in six cell lines with *β*-actin as a loading control. *D*, quantification of SOD1, SOD2, and catalase levels. Average relative values of SOD1, SOD2, and catalase were normalized to the average values of *β*-actin in various cell lines. The values for the latter are expressed as percentages of the average values for the control cell lines. The average of three independent determinations for each cell lines is shown. Graph details and symbols are explained in the legend to Figure 3. ROS, reactive oxygen species.

Mitochondria ROS (mito-ROS) plays the critical role in the physiological consequences (39). We next assessed ROS production in mutant cybrid cell lines *via* flow cytometry, comparing baseline staining intensity for each cell line to that upon oxidative stress in order to obtain a ratio corresponding to ROS generation (40, 41). Geometric mean intensity was recorded to measure the rate of mito-ROS of each sample. The relative levels of geometric mean intensity in each cell line were calculated to delineate the levels of mito-ROS in mutant and control cells. As shown in Figure 6, *A* and *B*, the levels of mitochondrial ROS in three mutant cybrids ranged from 114.1% to 141.9%, with an average of 125.0% of the mean value measured in three control cybrids. Furthermore, we examined the levels of three antioxidant enzymes: SOD2 in mitochondrial matrix, SOD1 and catalase in cytosol and mitochondrial intermembrane without and with treatment of the mutant and control cybrids with 100 nM of 10-methanesuflonate (MitoQ), a mitochondria-specific antioxidant to reduce oxidative stress (41, 42, 43). As shown in Figure 6, *C* and *D*, the marked increasing levels of these proteins were observed in the mutant cybrids. In particular, the average levels of SOD1, SOD2, and catalase in three mutant cybrids were 148.7%, 176.3%, and 159.4%, relative to the mean values measured in three control cybrids, respectively, whereas the levels of antioxidation enzymes in the mutant cybrids in the presence of MitoQ were comparable with those of control cybrids.

### Imbalanced mitochondrial dynamics

**Figure 7:**
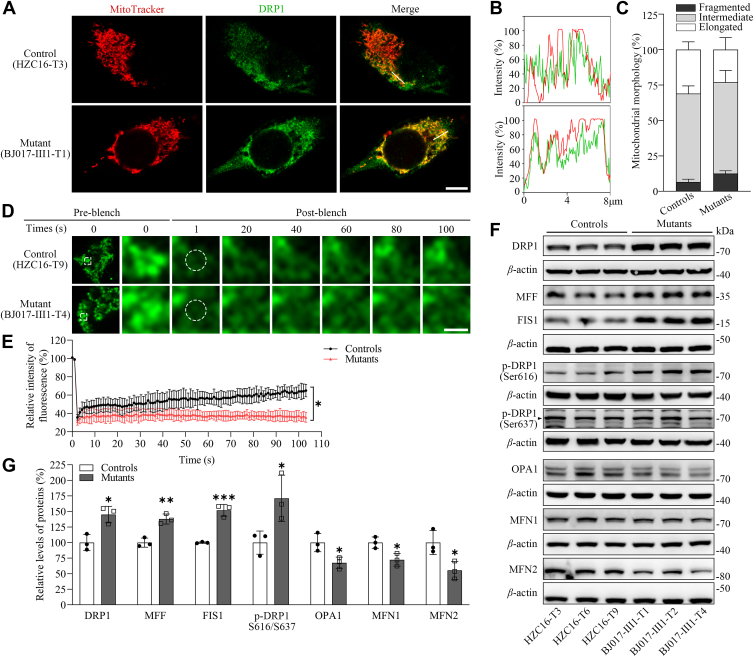
**Mitochondrial dynamics analysis.***A*, immunofluorescence analysis. The distributions of DRP1 from mutant BJ017-Ⅲ1-T1 and control HZC16-T3 cybrids was visualized by immunofluorescent labeling with DRP1 antibody conjugated to Alexa Fluor 488 (*green*) and MitoTracker (*red*) analyzed by confocal microscopy. DAPI-stained nuclei were identified by their *blue* fluorescence. Scale bars represent 10 μm. *B*, immunofluorescence colocalization analysis. Colocalization analysis of DRP1 and mitochondria in the control (HZC16-T3) and mutant (BJ017-Ⅲ1-T1) cell lines. *C*, quantification of mitochondrial morphologies, which were scored as follows: fragmented, mainly small and round; intermediate, mixture of round and shorter tubulated; and elongated, long, and higher interconnectivity. The percentage of cells with indicated mitochondrial morphologies was determined as a percentage of the total number of cells counted (100–200 cells per experiment). n = 3 independent experiments. *D*, time-lapse imaging of mitochondrial network connectivity using fluorescence recovery after photobleaching (FRAP). Mitochondria in control (HZC16-T9) and mutant (BJ017-Ⅲ1-T4) cybrids expressing Mito-GFP were photobleached with a 488 nm laser (50% ND filter) in a defined region of interest (ROI). Recovery of fluorescence intensity was monitored every 1 s for 100 s postbleach under live-cell imaging conditions. Scale bars represent 1 μm. *E*, quantification of mitochondrial network connectivity by FRAP analysis. Fluorescence recovery levels were normalized to prebleach baselines (mean intensity from two prebleach images). *F*, Western blot analysis of mitochondrial fission–associated proteins (DRP1, p-DRP1-Ser616, and p-DRP1-Ser637, MFF, and FIS1) and fusion-associated proteins (OPA1, MFN1, and MFN2) in six cell lines with *β*-actin as a loading control. *G*, quantification of mitochondrial fission–associated proteins (DRP1, MFF, FIS1, and p-DRP1S616/S637) and fusion-associated proteins (OPA1, MFN1, and MFN2). Three independent experiments were made for each cell line. Graph details and symbols are explained in the legend to Figure 3. DAPI, 4′,6-diamidino-2-phenylindole.

Defective stability and activity of OXPHOS complexes caused by the m.3733G>C mutation may affect the mitochondrial dynamics, which is achieved through continual fusion and fission (44, 45). To evaluate if the m.3733G>C mutation affected the mitochondrial dynamics, we examined the mitochondrial fission and fusion of mutant and control cybrids by immunofluorescence and Western blot analyses. We used immunofluorescence to assess the effect of m.3733G>C mutation on mitochondrial morphology and dynamics using cells staining with mitochondrial dye MitoTracker and labeling with antibody of fission-related protein DRP1. As illustrated in Figure 7, *A*–*C*, the mutant cells harboring the m.3733G>C mutation exhibited abnormal mitochondrial morphologies, characterized by significant increases in fragmented mitochondria and decreases in the elongated mitochondrial network, as compared with control cells. Furthermore, we performed fluorescence recovery after photobleaching (FRAP) experiments to examine the mitochondrial connectivity using cell lines transfected by mitoGFP. As shown in Figure 7, *D* and *E*, the levels of FRAP were decreased in the mutant cell lines, as compared with those in control cell lines. These indicated lower levels of functional connectivity of mitochondrial networks in the cells bearing the m.3733G>C mutation than those in control cells.

We then assessed the levels of three fission-related proteins (DRP1, FIS1, and MFF) and three fusion-related proteins (MFN1, MFN2, and OPA1) in mutant and control cell lines by Western blot analysis (44, 45). In particular, DRP1 is the major profission protein whose phosphorylation at Ser616 or Ser637 promotes and inhibits mitochondrial fission process, respectively (46, 47). As shown in Figure 7, *F* and *G*, the mutant cells revealed marked increasing levels of DRP1 than those in control cells. The levels of DRP1 with phosphorylation at Ser616 or Ser637 in mutant cybrids were increased and decreased, as compared with those in control cybrids, respectively. Furthermore, average levels of MFF and FIS1 in three mutant cell lines were 137.9% and 151.8% of the average values measured in three control cell lines, respectively. This indicated that the m.3733G>C mutation facilitated mitochondrial fission. As shown in Figure 7, *F* and *G*, the average levels of OPA1, MFN1, and MFN2 in mutant cell lines were 67.4%, 72.3%, and 55.3% of the average values measured in three control cell lines, respectively. After the treatment of cells with MitoQ, the levels of DRP1, FIS1, MFF, OPA1, MFN1, and MFN2 in the mutant cybrids were comparable with those in three control cybrids (Fig. S1). These data indicated that the m.3733G>C mutation resulted in the mitochondrial dynamic imbalance toward fission.

### Impaired mitophagy

**Figure 8:**
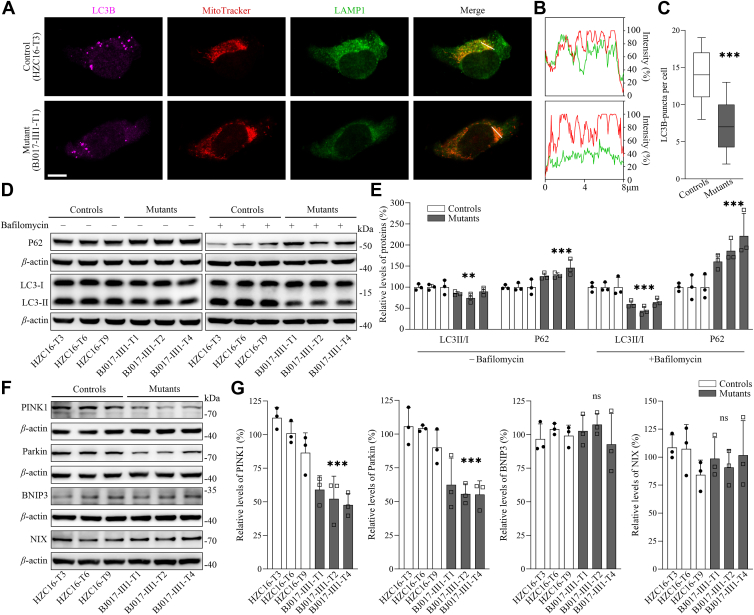
**Analysis of mitophagy.***A*, immunofluorescence analysis. The distributions of LC3B and LAMP1 from mutant BJ017-Ⅲ1-T1 and control HZC16-T3 cell lines were visualized by immunofluorescent staining with mitochondrial dye MitoTracker (*red*) and labeling with LAMP1 and LC3B. Scale bars represent 10 μm. *B*, immunofluorescence colocalization analysis. Colocalization analysis of LAMP1 and mitochondria in the control (HZC16-T3) and mutant (BJ017-Ⅲ1-T1) cell lines. *C*, number of LC3B puncta per cell, in the controls and mutant cell lines (100–200 cells per experiment). *D*, Western blot analysis for autophagic-related proteins: LC3B and P62 in the presence and absence of 50 nM bafilomycin in six cell lines with *β*-actin as a loading control. *E*, quantification of autophagy-related proteins (LC3II/I and P62) in the mutant and control cell lines. *F*, Western blot analysis of four mitophagy-related proteins (PINK1, Parkin, BNIP3, and NIX) in various cell lines. *G*, quantification of PINK1, Parkin, BNIP3, and NIX. Three independent determinations were done in each cell line. Graph details and symbols are explained in the legend to Figure 3.

The alterations in OXPHOS affected the mitophagic removal of damaged mitochondria (42, 48, 49, 50). Mitophagy is a specific form of autophagy that selectively removes damaged mitochondria by autophagosomes and their subsequent catabolism by lysosomes (50). To investigate if the m.3733G>C mutation regulated the autophagy, the autophagic states of various mutant and control cybrid cell lines were analyzed using both immunofluorescence and Western blot assays. As shown in Figure 8, *A*–*C*, the mutant cell lines exhibited decreased levels of LAMP1 in the mitochondria and light chain 3B (LC3B)-puncta, compared with those in control cells, indicating that the m.3733G>C mutation impaired autophagy. We then measured the level of two autophagy markers: microtubule-associated protein 1A/1B light chain 3B and sequestosome 1 (SQSTM1/p62) in various cell lines using a Western blot analysis. LC3-II is recleaved by cysteine protease (Atg4B) following completion of the autophagosome, and recycled, whereas SQSTM1/p62, one of the best-known autophagic substrates, interacts with LC3 to ensure the selective delivery of these proteins into the autophagosome (51). As shown in Figure 8, *D* and *E*, the average levels of LC3II/I and P62 in three mutant cybrids were 82.9% and 133.2% of the mean values measured in three control cybrids, respectively. The treatment of cells with bafilomycin, an inhibitor of late phase of autophagy, elevated the level of P62 but reduced the level of LC3II/I in the mutant cybrids (52). We then examined the levels of Parkin and PINK1 involved in ubiquitin-dependent mitophagy, BNIP3 and NIX involved in ubiquitin-independent pathways (48, 49, 50). As shown in Figure 8, *F* and *G*, the average levels of PINK1, Parkin, BNIP3, and NIX in mutant cybrids were 52.9%, 57.7%, 101%, and 97.1% of the mean values measured in control cybrids, respectively. These highlighted the impact of LHON-associated ND1 mutation on mitophagy *via* ubiquitin-dependent pathway.

### Promoting apoptosis

**Figure 9:**
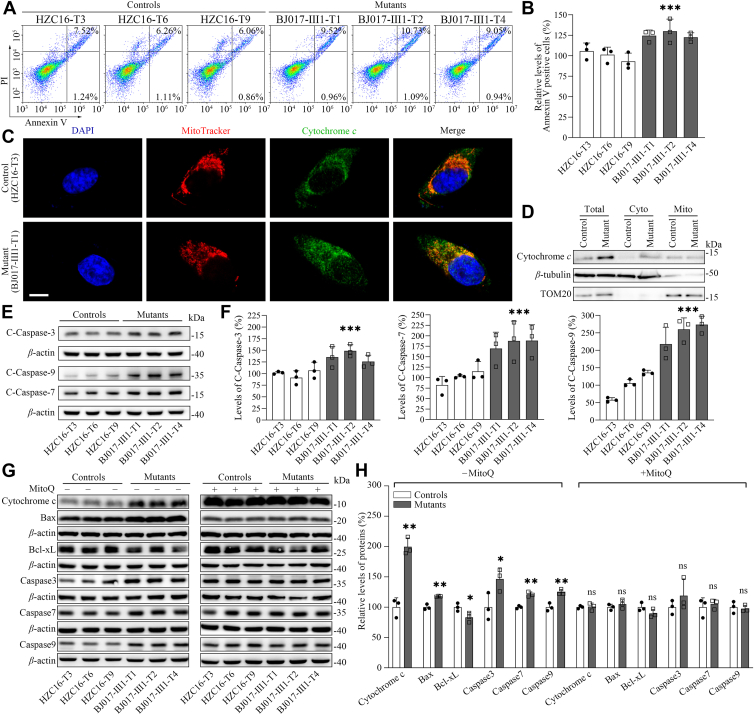
**Analysis of apoptosis.***A*, Annexin V/propidium iodide (PI) apoptosis assay by flow cytometry. Cells were harvested and stained with Annexin V and 1 μl of PI. The percentage of Annexin V-positive cells was then assessed. *B*, relative Annexin V-positive cells from various cell lines. Three independent determinations were done in each cell line. *C*, immunofluorescence analysis. The distributions of cytochrome *c* from mutant BJ017-Ⅲ1-T1 and control HZC16-T3 cybrids were visualized by immunofluorescent labeling with cytochrome *c* antibody conjugated to Alexa Fluor 488 (*green*) and MitoTracker (*red*) analyzed by confocal microscopy. DAPI-stained nuclei were identified by their *blue* fluorescence. Scale bars represent 10 μm. *D*, the levels of cytochrome *c* in cytosol in mutant and control cell lines were measured by fractioning the cells into mitochondrial and cytosolic fractions and Western blot analysis using antibodies of cytochrome *c*, TOM20 for mitochondrial protein, and *β*-tubulin for cytosolic protein. Cyto, cytosol; Mito, mitochondria; Total, total cell lysate. *E*, Western blotting analysis. Cellular proteins (20 μg) from various cell lines were electrophoresed, electroblotted, and hybridized with several apoptosis-associated protein antibodies cleaved caspases 3, 7, and 9 with *β*-actin as a loading control. *G*, Western blotting analysis. Cellular proteins (20 μg) from various cell lines were electrophoresed, electroblotted, and hybridized with several apoptosis-associated protein antibodies: cytochrome *c*, BAX, Bcl-xL, uncleaved caspases 3, 7, and 9 in the presence and absence of 100 nM MitoQ with *β*-actin as a loading control. *F* and *H*, quantification of apoptosis-associated proteins: cytochrome *c*, BAX, Bcl-xL, cleaved/uncleaved caspases 3, 7, and 9. Three independent experiments were made for each cell line. Graph details and symbols are explained in the legend to Figure 3. DAPI, 4′,6-diamidino-2-phenylindole.

Deficient activities of OXPHOS have been linked to the protection against certain apoptotic stimuli (21, 22, 23). The effect of m.3733G>C mutation on apoptotic process was examined by Annexin V/propidium iodide (PI)–based flow cytometry for cellular apoptosis, immunofluorescence, and Western blot assays. As shown in Figure 9, *A* and *B*, the average ratios of Annexin-V-positive cells in mutant cell lines harboring the m.3733G>C mutation were 125.7% of relative to the mean values measured in control cell lines. As shown in Figure 9*C*, the immunofluorescence patterns of double-labeled cells with rabbit monoclonal antibody specific for cytochrome *c* and MitoTracker exhibited significantly increased levels of cytochrome *c* in the mutant cybrids, compared with those in control cybrids. The levels of cytochrome *c* in cytosol in mutant and control cell lines were further assessed by fractioning the cells into mitochondrial and cytosolic fractions and Western blot analysis. As shown in Figure 9*D*, the levels of cytochrome *c* in the mutant cell lines were markedly increased in the total cellular and cytosolic fractions, as compared with those in control cell lines. The effect of the m.3733G>C mutation on the apoptotic process was further assessed to examine the levels of apoptotic proteins (BAX, caspases 9, 7, and 3) and antiapoptotic protein (Bcl-xL) using Western blot analysis (53, 54, 55, 56). As shown in Figure 9, *E* and *F*, the mutant cybrids exhibited 136.7%, 181.9%, and 250.5% levels of cleaved caspases 3, 7, and 9, as compared with those in control cybrids. As shown in Figure 9, *G* and *H*, the average levels of BAX, Bcl-xL, uncleaved caspases 3, 7, and 9 in three mutant cell lines were 117.8%, 83.1%, 146.4%, 121.1%, and 124.7% of the average values measured in three control cell lines, respectively. The treatment of cells with MitoQ made the levels of these proteins in the mutant cybrids to comparable levels of these in control cybrids. These results indicated that the m.3733G>C mutation promoted apoptotic process.

## Discussion

In the present study, we have investigated the molecular mechanism of LHON-associated ND1 3733G>C mutation. The incidences of the m.3733G>C mutation were 0.17% in the cohorts of 1793 Chinese probands with LHON. The occurrence of the m.3733G>C mutation in four genetically unrelated Chinese and Germany pedigrees and affected by LHON and differing considerably in their mtDNA haplotypes (G2a1, R11a, D4, and J1c3e1) strongly indicated that this mutation is involved in the pathogenesis of LHON (57). However, two of three Chinese pedigrees carrying the m.3733G>C mutation did not have a family history of optic neuropathy. The extremely lower penetrance of LHON in these families carrying the m.3733G>C mutation was comparable with those in Chinese families bearing only one LHON-associated mtDNA mutation(s), including m.3394T>C, m.3460G>A, m.3866T>C, m.11778G>A, m.12338T>C, or m.14484T>C mutations (17, 18, 21, 22, 23, 58). Furthermore, the m.3733G>A (p.E143K) mutation was found in three European families (mtDNA haplotypes H10, X2b) and one Korean pedigree with LHON (57, 59, 60). These highlighted the involvement of m.3733G>A-C (p.E143K-Q) mutation in the pathogenesis of LHON.

The m.3733G>C (E143Q) mutation changed a highly conserved glutamic acid at position 143 with glutamine in ND1, which is an essential subunit of complex I (27, 60). To evaluate the impact of the E143Q mutation on the structure and function of ND1, we performed MD simulation analysis. Based on the crystal structure of mammalian complex I structure (61), there are the interactions between residues E143 and S110 and Y114 or between S141 and W290 in WT ND1 structure. The substitution of glutamic acid (E143) with glutamine (Q143) made the replacement of a carboxyl group with an amide group and destabilized these interactions between residues E143 and S110/Y114 or between S141 and W290. Strikingly, the alterations introduced the new interactions between Q143 and E192/S109 in the ND1. The effect of E143Q mutation on structure of ND1 was evidenced by the reduced levels of ND1 observed in cybrid cell lines bearing the m.3733G>C mutation. The decreased levels of ND1 may be due to lower expression or instability of mutant ND1 molecule but not proteostasis stress.

In fact, complex I subunits are organized into six modules, Q, ND1, ND2, ND4, ND5, and N, with the help of specific assembly factors that begin to assemble separately and ultimately combine to form the complex I holocomplex in the inner membrane of mitochondria (60, 61, 62). Thus, we hypothesized that the m.3733G>C mutation altered the assembly, stability, and activity of complex I. The mutated ND1 indeed perturbed the assembly of supercomplexes and complex I, as in the case of m.3394T>C, m.3460G>A, m.11778G>A, m.12338T>C, and m.14484T>C (19, 21, 22, 23). Both lower level of ND1 and altered assembly of complex I were responsible for 22% reductions in the activity of complex I observed in cybrids carrying the m.3733G>C mutation. Furthermore, the m.3733G>C mutation yielded reduced rates in the basal OCR, ATP-linked OCR, proton-linked OCR, and maximal OCR in mutant cybrid cell lines. These respiratory deficiencies resulted in impairing ATP synthesis and excessive production of oxidative reactive species required for vision function. The contribution of assembly of complex I or increase ROS to the downstream events were evidenced by reducing m.3733G>C mutation–induced oxidative stress by treating cells with MitoQ, a mitochondria-specific antioxidant (43). These data highlighted that the defective activity of complex I in the mutant cell lines carrying the m.3733G>C mutation plays an important role in producing mitochondrial dysfunction for the manifestation of LHON phenotype.

These m.3733G>C mutation–induced dysfunction may reprogram mitochondrial integrity *via* their quality control by mitochondrial dynamics and mitophagy and cellular homeostasis *via* autophagy and apoptosis. The effect of m.3733G>C mutation on mitochondrial quality control was evidenced by elevating mitochondrial localization of DRP1, decreasing network connectivity and abnormal morphologies, characterized by increasing fragmented mitochondria and decreasing elongated mitochondria. These fragmental mitochondria resulted in imbalance of mitochondrial dynamics toward fission, supported by upregulating fission-related genes, DRP1, FIS1, and MFF, but downregulating fusion-related proteins, OPA1, MFN1, and MFN2 in the mutant cybrids (44, 45). Alternatively, these fragmental mitochondria are likely to be targeted for mitophagy to dispose of damaged mitochondria (49, 50). In this study, we demonstrated that the m.3733G>C mutation downregulated the ubiquitin-dependent mitophagy, evidenced by reducing levels in the PINK1 and Parkin, and did not affect ubiquitination-independent mitophagy, supported by no changes in the expression of BNIP3 and NIX in the mutant cells. These results were consistent with those in cell bearing LHON-associated m.3460G>A and m.14484T>C mutation (63, 64) but in contrast with those in the cells carrying LHON-associated tRNA^Ala^ 5587A>G mutation, deafness-associated 12S rRNA 1555A>G, tRNA^Phe^ 593T>C, and 7516delA mutation (20, 65, 66, 67). These discrepancies may reflect the defects in the sole ND1 subunit arising from m.3733G>C or m.3460G>A mutations but multiple subunits because of m.593T>C, m.1555A>G, m.5587A>G, and 7516delA mutations.

The m.3733G>C mutation–induced deficiencies regulated the cellular homeostasis *via* autophagy and apoptosis. The impaired autophagy induced by m.3733G>C mutation was evidenced by decreased levels of LAMP1 in the mitochondria and LC3B-puncta in the mutant cells using immunofluorescence assay. The effect of m.3733G>C mutation on autophagy was further supported by decreased levels of LC3 but increased levels of P62 in mutant cells carrying the m.3733G>C mutation. Alternatively, the m.3733G>C mutation–induced deficiencies promoted intrinsic apoptotic process for the removal of damaged cells. In this study, we demonstrated that the m.3733G>C mutation upregulated the apoptotic process, evidenced by markedly increased levels of Annexin V intensity and release of cytochrome *c* into the cytosol. The release of cytochrome *c* promotes the activation of caspase-3, -7, and -9, which subsequently initiates cell death. The impact of m.3733G>C mutation on the intrinsic apoptotic process was further supported by decreased levels of antiapoptotic protein BcL-XL. These findings demonstrated that the m.3733G>C mutation–induced deficiencies reprogram autophagy and apoptosis pathways to maintain cell integrity.

In summary, our findings demonstrate the impact of LHON-associated ND1 3733G>C mutation on mitochondrial and cellular integrity. The 3733G>C (E143Q) mutation perturbed the structure and function of ND1, thereby altering the assembly and activity of complex I. As a result, this respiratory deficiency gave rise to the decrease of mitochondrial ATP production and the increasing production of oxidative reactive species. The m.3733G>C mutation–induced deficiencies regulated the mitochondrial integrity by causing mitochondrial dynamic imbalance toward fission and downregulating the ubiquitin-dependent mitophagy. Finally, the m.3733G>C mutation–induced deficiencies impacted the homeostasis of cells *via* promoting autophagy and intrinsic apoptosis. The broad effect of m.3733G>C mutation on mitochondrial and cellular integrity may regulate various aspects of vision function, thereby being critical for the pathogenesis of LHON.

## Experimental procedures

### Families and subjects

Three Chinese pedigrees with LHON were recruited from a large cohort of 1793 Chinese probands with LHON, as described previously (15, 22, 23). This study was in compliance with the Declaration of Helsinki. Informed consent, blood samples, and clinical evaluations were obtained from all participating family members, under protocols approved by the Ethic Committees of Zhejiang University. A comprehensive history and physical examination for these participating subjects were performed at length to identify both personal or family medical histories of visual impairment and other clinical abnormalities. The ophthalmic examinations of probands and other members of these families were conducted as detailed previously (16, 17, 18). A total of 485 control DNA samples were obtained from vision normal Han Chinese subjects from same area.

### mtDNA analysis

The sequence analysis of entire mitochondrial genomes of three Chinese probands carrying the m.3733G>C mutation was determined as detailed elsewhere (68). The resulting sequence data were compared with the updated consensus Cambridge sequence (GenBank accession number: NC_012920) (69). The entire mtDNA sequences of three Chinese pedigrees and one control subject were assigned to the mitochondrial haplogroups using the nomenclature as described previously (30). An analysis for the presence and levels of m.3733G>C mutations in mutant and control cell lines was carried out as described previously (57).

### Cell lines and culture conditions

Lymphoblastoid cell lines were immortalized by transformation with the Epstein–Barr virus, as described elsewhere (70). Cell lines derived from the affected matrilineal relative (BJ017-III-3, male, 20 years) carrying the m.3733G>C mutation and one control subject (HZC16, male, age 22 years) lacking the mutation but belonging to the same mtDNA haplogroup D4 were grown in the RPMI1640 medium (Thermo Fisher Scientific) supplemented with 10% fetal bovine serum (FBS). The 143B.TK^–^ cell line was grown in Dulbecco’s modified Eagle’s medium (containing 4.5 mg/ml glucose and 0.11 mg/ml pyruvate), supplemented with 100 μg/ml bromodeoxyuridine and 5% FBS. The mtDNA-less *ρ*^0^206 cell line, derived from 143B.TK^–^ was grown under the same conditions as the parental line, except for the addition of 5 mg/ml uridine (28). Transformation by cytoplasts of mtDNA-less *ρ*^0^206 cells was performed using immortalized lymphoblastoid cell lines, as detailed previously (28, 29). The cybrids derived from each donor cell line were analyzed for the presence and level of the m.3733G>C mutation and mtDNA copy numbers as detailed elsewhere (28, 57). Three cybrids derived from each donor cell line bearing homoplasmic m.3733G>C mutation and similar mtDNA copy numbers were used for the following biochemical characterization. All cybrid cell lines were maintained in the same medium as the 143B.TK^–^ cell line.

### MD simulations

MD simulations were carried out to evaluate the effect of m.3733G>C mutation. To balance computational feasibility and simplify model, only ND1 was extracted from the complex I structure for simulations. The coordinates of WT ND1 were derived from the cryo-EM structure of human mitochondrial respiratory complex I (PDB entry: 5XTD) (27). The positively charged residue K262 was treated as a deprotonated form, and the terminal residues were set to a charged state. The coordinates of E143Q mutant were generated using the mutagenesis module of PyMOL. Subsequently, CHARMM-GUI Membrane Builder (http://www.charmm-gui.org) was employed to construct protein-membrane systems (68). Both WT and mutant ND1 were embedded into a pure palmitoyl-oleoyl-phosphatidylcholine (POPC) bilayer, which were fully immersed in a simple point charge water model. Na^+^ and Cl^-^ were added to maintain a constant concentration (50 mmol/l) and neutralize the excess negative charge.

MD simulations were performed using GROMACS 4.5.5 package with CHARMM36 force field (71, 72). Initially, energy minimization was performed to eliminate unfavorable steric clashes, with positional restraints applied to all heavy atoms of protein and phosphorus atoms of POPC. The energy-minimized structures were prepared for subsequent equilibration steps. For each system, six equilibration steps were carried out, gradually reducing positional constraints on the backbone (from 4000 to 50 kJ/mol/nm^2^) and side chain (from 2000 to 0 kJ/mol/nm^2^) atoms of protein, as well as on the phosphorus atoms of POPC (from 1000 to 0 kJ/mol/nm^2^). Each system was equilibrated in two 25 ps NVT ensembles, heating from 0 to 310 K with a time step of 1 fs. Following this, a series of equilibration steps totaling 162.5 ns were performed at NPT ensemble to fully equilibrate the lipid bilayer and the solvent. The temperature was maintained at 310 K using the Nose–Hoover thermostat algorithm (73, 74, 75), whereas pressure was coupled to 1 bar using the Parrinello–Rahman algorithm (76, 77). Electrostatics were calculated using the particle-mesh Ewald method (78). Finally, the MD productions were conducted for 500 ns with a time step of 2 fs. All coordinates were saved every 10 ps. The trajectory of the WT MT-ND1 was derived from previous investigation (19).

The analysis of trajectories for both the WT and mutant systems was conducted utilizing Gromacs 4.5.5 packages. The RMSD fluctuating curves were generated using R, version 3.4.1. To visualize the trajectory of each system, VMD (79) was employed, whereas PyMOL was used to create all figures of molecular structure.

### Western blot analysis

Western blotting analysis was carried out as detailed previously (38). About 20 μg of total cellular proteins were electrophoresed through 10% Bis–Tris SDS-polyacrylamide gels and then transferred to a polyvinyl difluoride membrane. The antibodies used for this investigation were from Abcam (ND3 [ab192306] and P62 [ab56416]), Proteintech Group (ND1 [197031-AP], TOM20 [11802-1-AP], *β*-actin [66009-1-Ig], CO2 [55070-1-AP], ATP8 [26723-1-AP], ATP6 [68442-1-Ig], CLPP [15698-1-AP], AFG3L2 [14631-1-AP], cytochrome *c* [10993-1-AP], BAX [50599-2-Ig], Caspase3 [19677-1-AP], Caspase7 [27155-1-AP], Caspase9 [10380-1-AP], OPA1 [27733-1-AP], FIS1 [10956-1-AP], MFN1 [13798-1-AP], MFF [17090-1-AP], MFN2 [12186-1-AP], DRP1 [12957-1-AP], and *β*-Tubulin [10094-1-AP]), ABclonal Technology (ND6 [A17991], NDUFS2 [A21117], NDUFA8 [A12118], NDUFA13 [A3782], SOD1 [A12537], SOD2 [A19576], Catalase [A11220], cleaved caspase3 [A27145], cleaved caspase7 [A23154], and Parkin [A0968]), Signalway antibody (ND4L [460760]), Cell Signaling Technology (Bcl-Xl [2764T], cleaved caspase9 [9505T], and LC3B [43566S]), Sangon Biotech (BNIP3 [D121876]), Santa Cruz (NIX [sc-166332]), and Abcepta (PINK1 [AW5456]). Peroxidase AffiniPure goat antimouse IgG and goat anti-rabbit IgG (Beyotime Biotechnology) were used as a secondary antibody, and protein signals were detected using the ECL system (Millipore). Quantification of density in each band was performed as detailed previously (38).

### Assays of activities of OXPHOS complex

BN-PAGE and in-gel activity assays were performed using mitochondrial proteins isolated from mutant and control cybrid cell lines, as detailed elsewhere (34, 35, 36). Samples containing 30 mg of total cellular proteins were separated on 3% to 12% Bis–Tris Native PAGE gel. The primary antibodies used for this experiment were from Proteintech Group (SDHB [10620-1-AP] and COX5A [11448-1-AP]) and ABclonal Technology (NDUFA10 [A10123] and VDAC1 [A19707]). Alkaline phosphatase–labeled goat anti-mouse IgG and goat anti-rabbit IgG (Beyotime) were used as secondary antibodies, and protein signals were detected using the BCIP/nitroblue tetrazolium (NBT) Alkaline Phosphatase Color Development Kit (Beyotime).

The in-gel activity assay was performed as detailed elsewhere (36). Briefly, the Native PAGE gels were prewashed in ice-cold water and then incubated with the substrates of complex I (NBT, NADH), complex II (sodium succinate, NBT, and phenazine methosulfate), and complex Ⅳ (3,3′-diaminobenzidine and cytochrome *c*). The gels were then incubated at 37 °C overnight. After the reaction was stopped with 10% acetic acid, the gels were washed extensively in water and scanned to visualize the activities of the respiratory chain complexes.

### Measurements of oxygen consumption

The OCR in various cybrid cell lines was measured with a Seahorse Bioscience XF-96 extracellular flux analyzer (Seahorse Bioscience), as detailed previously (37, 38). Cells were seeded at a density of 2 × 10^4^ cells per well on Seahorse XF96 polystyrene tissue culture plates (Seahorse Bioscience). Inhibitors were used at the following concentrations: oligomycin (1.5 mM), carbonyl cyanide p-(trifluoromethoxy) phenylhydrazone (0.8 mM), antimycin A (1.5 mM), and rotenone (3 mM), respectively.

### ATP measurements

The cellular and mitochondrial ATP levels were analyzed by a Cell Titer-Glo luminescent cell viability assay kit (Promega) according to the modified procedures of the manufacturer, as described previously (38).

### ROS measurement

The MitoSOX Red Mitochondrial Superoxide Indicator (Thermo Fisher) was used for ROS measurements following the manufacturer's instructions, as detailed previously (40, 41).

### Immunofluorescence analysis

Immunofluorescence experiments were undertaken as described elsewhere (66, 67). Cells were cultured on cover glass slips (ThermoFisher), fixed in 4% formaldehyde for 15 min, permeabilized with 0.2% Triton X-100, blocked with 5% FBS for 1 h, and immune-stained with DRP1, cytochrome *c*, LC3B antibodies overnight at 4 °C, respectively. The cells were then incubated with Alexa Fluor 594 goat anti-rabbit IgG, Alexa Fluor 488 goat anti-rabbit IgG, and Alexa Fluor 488 goat anti-mouse IgG (ThermoFisher), stained with 4′,6-diamidino-2-phenylindole (ThermoFisher) for 15 min, and mounted with Fluoromount (Sigma–Aldrich). Cells were examined using a confocal fluorescence microscope (Olympus Fluoview FV1000) with three lasers (excitation/emission = 550/570, 492/520, and 358/461 nm).

### FRAP analysis

Mito-GFP–expressing cells were used for FRAP analyses at 48 to 72 h after transfection. To evaluate mitochondrial connectivity in Mito-GFP–expressing cells, GFP signals were photobleached with a 488 nm laser (50% ND filter) for 0.2 s. The bleached region of interest was monitored for recovery by imaging with the same laser (0.1% ND filter) every 1 s for 100 s. Mito-GFP signal intensity was normalized to the mean intensity of the region of interest calculated from two prebleached images, which served as baseline, using the same laser (0.1% ND filter).

### Annexin V/PI apoptosis assay by flow cytometry

For discrimination of apoptotic and nonapoptotic cells by Annexin V/PI staining, cells were harvested and stained with Annexin V and 1 ml of PI (V13242; Thermo Fisher Scientific) according to the manufacturer’s instruction, as described elsewhere (20). Each sample was detected by NovoCyte (Agilent Technologies) and analyzed using NovoExpress software (Agilent Technologies).

### Statistical analysis

All statistical analyses were performed using GraphPad Prism (version 9.00; GraphPad Software, Inc) for statistical analysis to compare outcomes using a two-tailed unpaired Student’s *t* test. *p* Values of less than 0.05 were considered to be statistically significant. ∗*p* < 0.05; ∗∗*p* < 0.01; ∗∗∗*p* < 0.001; ns, not significant.

## Data availability

The authors declare that all relevant data of this study are available within the article or from the corresponding author (gminxin88@zju.edu.cn) upon reasonable request.

## Supporting information

This article contains supporting information (Fig. S1, Tables S1, and S2).

## Conflict of interest

The authors declare that they have no conflicts of interest with the contents of this article.
