# Supplementary material for: Leber's hereditary optic neuropathy–associated ND1 3733G>C mutation ameliorates the mitochondrial quality control and cellular homeostasis
Source: J Biol Chem. 2025 Jul 8;301(8):110464. doi: 10.1016/j.jbc.2025.110464 (PMC12340437; doi:10.1016/j.jbc.2025.110464)
Supplement: Supplementary File [file mmc1.pdf]

SUPPLEMENTARY INFORMATION

Supplementary Figure S1

Supplementary Table S1

Supplementary Table S2

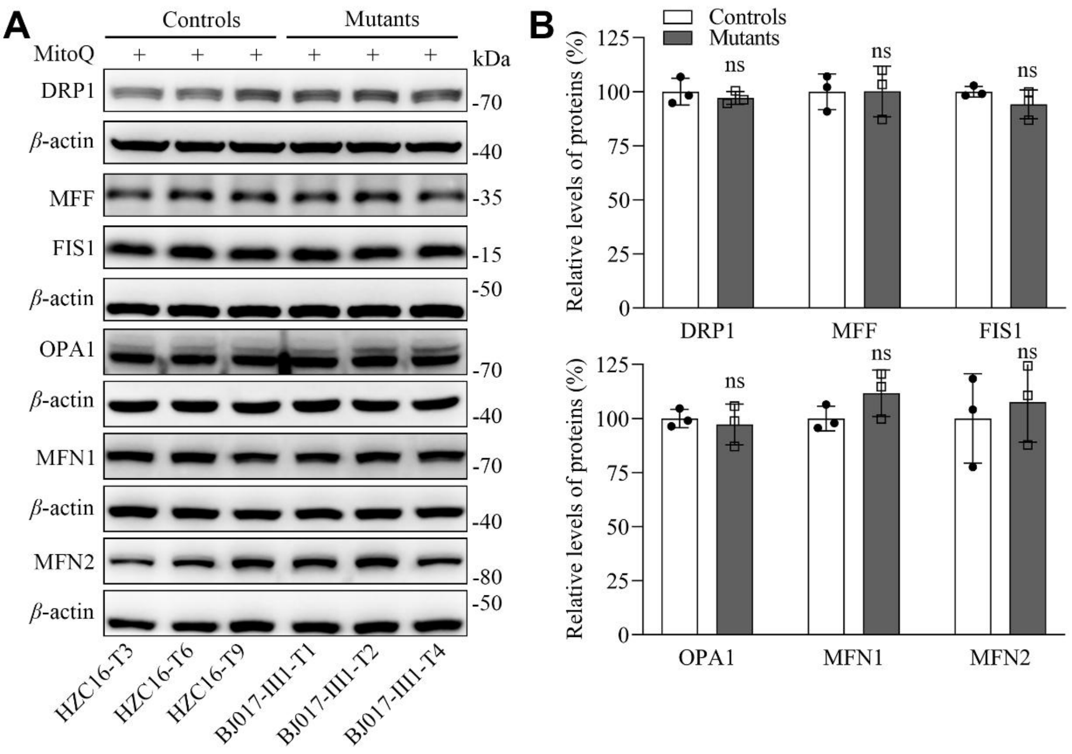

Supplementary Figure S1. Mitochondrial dynamics analysis of mutant and control cell lines in the presence of 100 nM MitoQ. (A) Western blot analysis of mitochondrial fission-associated proteins (DRP1, MFF, and FIS1) and fusion-associated proteins (OPA1, MFN1, and MFN2) in the presence of 100 nM MitoQ in six cell lines with *β*-actin as a loading control. (B) Quantification of mitochondrial fission-associated proteins (DRP1, MFF and FIS1) and fusion-associated proteins (OPA1, MFN1, and MFN2). The calculations were based on three independent determinations in each cell line. The error bars indicate two standard errors of the means. *P* indicates the significance, according to the student t-test of the differences between mutant and control cell lines.

Supplementary Table S1. Summary of clinical data for members of three vision-impaired Chinese pedigrees carrying the m.3733G>C mutation

Subject	Sex	Age of test (years)	Age of onset (years)	Visual acuity, right	Visual acuity, left	Level of visual impairment
BJ005-I-1	M	71	NA	1.0	0.8	Normal
BJ005-I-2	F	69	NA	1.0	1.0	Normal
BJ005-II-1	M	49	NA	1.5	1.0	Normal
BJ005-II-2	F	49	NA	0.8	0.6	Normal
BJ005-II-3	F	45	NA	1.0	1.0	Normal
BJ005-II-4	F	42	NA	1.0	0.6	Normal
BJ005-II-5	M	40	NA	1.5	1.2	Normal
BJ005-II-6	M	36	NA	1.5	1.0	Normal
BJ005-II-7	F	35	NA	1.0	1.0	Normal
BJ005-III-1	M	20	NA	1.0	0.7	Normal
BJ005-III-2	M	15	NA	1.0	0.9	Normal
BJ005-III-3	M	11	11	0.1	0.2	Mild
BJ005-III-4	F	9	NA	0.8	1.0	Normal
BJ005-III-5	F	7	NA	0.9	0.6	Normal
BJ005-III-6	M	10	NA	1.5	1.5	Normal
BJ005-III-7	F	6	NA	1.0	1.0	Normal
BJ017-I-1	M	84	NA	1.0	0.5	Normal
BJ017-I-2	F	83	NA	0.6	0.8	Normal
BJ017-II-1	M	63	NA	1.0	1.0	Normal
BJ017-II-2	F	60	NA	0.8	0.8	Normal
BJ017-II-3	M	61	NA	0.8	1.0	Normal
BJ017-II-4	F	58	57	0.1	0.1	Mild
BJ017-II-5	M	55	NA	1.0	1.0	Normal
BJ017-III-1	M	23	23	0.2	0.1	Mild
BJ017-III-2	M	18	NA	1.0	1.5	Normal
BJ017-III-3	M	22	20	0.1	0.1	Moderate
XT239-I-1	M	62	NA	0.5	0.5	Normal
XT239-I-2	F	60	NA	1.0	0.8	Normal
XT239-II-1	M	42	NA	0.6	0.8	Normal
XT239-II-2	F	42	NA	1.0	1.0	Normal
XT239-II-3	M	38	NA	1.0	1.0	Normal
XT239-II-4	F	37	NA	1.5	1.5	Normal
XT239-II-5	M	30	NA	0.6	0.6	Normal
XT239-II-6	M	28	NA	0.6	0.8	Normal
XT239-II-7	M	25	NA	0.7	0.8	Normal
XT239-II-8	F	24	NA	0.9	1.0	Normal
XT239-III-1	M	19	18	0.2	0.08	Moderate
XT239-III-2	M	13	NA	1.0	1.0	Normal
XT239-III-3	F	4	NA	1.0	1.5	Normal

The degree of visual impairment was defined according to the visual acuity as follows: normal >0.3, mild =0.3-0.1; moderate<0.1-0.05; severe<0.05-0.02; and profound <0.02. M, Male; F, Female.

Supplementary Table S2. mtDNA variants in three probands carrying the m.3733G>C mutation and one control subject in three Chinese families with LHON

Gene	Position	Replace	AA change	Conservation index (%)	BJ005-III-3	BJ017-III-1	XT239-III-1	HZC16 (control)
D-loop	73	A-G			G	G	G	G
	152	T-C			C	C		C
	207	G-A			A			
	235	A-G			G			
	249	A-G					G	
	263	A-G				G	G	G
	310	T-C/ TC /CTC			TC	CTC	C	CTC
	489	T-C				C	C	C
	514	CA-Del						
	523	A-Del			A-Del			
	524	C-Del			A-Del			
	16093	T-C					C	
	16111	C-G						G
	16129	G-A				A		A
	16223	C-T			T	T	T	T
	16256	C-T				T		
	16290	C-T			T			
	16298	T-C					C	
	16319	G-A			A			
	16327	C-T					T	
	16362	T-C			C	C		C
	16519	T-C				C	C	
12S rRNA	663	A-G		53	G			
	750	A-G		100	G	G	G	G
	1438	A-G		100	G	G	G	G
16S rRNA	1736	A-G		97.29	G			
	2706	A-G		57.14	G	G	G	G
	3010	G-A		50		A		A
	3106	C-Del		6				
	3206	C-T		31		T		T
ND1	3733	G-C	Glu143Gln	100	C	C	C	
ND2	4824	A-G	Thr119Ala	58	G			
	4883	C-T		47.12				T
	5178	C-A	Leu237Met	57.14		A		A
	5262	A-G	Ala265Thr	38			G	
$tRNA^{Cys}$	5821	G-A		33			A	
CO1	7028	C-T		47.06				T

ATP8	8414	C-T	Leu17Phe	71.43		T		T
	8459	A-G	Asn32Asp	44	G			
ATP6	8701	A-G	Thr59Ala	64.29		G		G
	8794	C-T	His90Tyr	69	T			
	8860	A-G	Thr112Ala	78.57	G	G		G
	9017	T-C	Ile164Thr	88	C			
ND3	10398	A-G	Thr114Ala	35.71		G	G	G
$tRNA^{Arg}$	10410	T-C		16		C		
ND4	11084	A-G	Thr109Ala	94	G			
	11447	G-A	Val230Met	81			A	
$tRNA^{His}$	12192	G-A		44	A			
ND6	14318	T-C	Asn119Ser	50			C	
Cytb	14766	C-T	Thr7Ile	50	T	T	T	
	14927	A-G	Thr61Ala	75	G			
	14979	T-C	Ile78Thr	44		C		C
	15326	A-G	Thr194Ala	64.29	G	G	G	G

[a] Conservation index (CI) was calculated by comparing the human mtDNA variants with other 16 vertebrates: *Bos Taurus, Cebus albifrons, Gorilla gorilla, Hylobates lar, Lemur catta, Macaca mulatta, Macaca sylvanus, Mus musculus, Nycticebus coucang, Pan paniscus, Pan troglodytes, Pongo pygmaeus, Pongo abelii, Papio hamadryas, Tarsius bancanus,* and *Xenopus laevis*.

[b]See the online mitochondrial genome database http://www.mitomap.org and http://www.genpat.uu.se/mtDB.
